# Long-Term Efficacy of Posterior Neurectomy in Anterior Cutaneous Nerve Entrapment Syndrome

**DOI:** 10.3389/jaws.2024.13508

**Published:** 2024-10-03

**Authors:** Tom ten Have, Monica L. Y. E. Jacobs, Marc R. M. Scheltinga, Willem A. R. Zwaans, Rudi M. H. Roumen

**Affiliations:** ^1^ Department of Surgery, Máxima Medical Centre, Veldhoven, Netherlands; ^2^ SolviMáx, Centre of Excellence for Abdominal Wall and Groin Pain, Máxima Medical Centre, Eindhoven, Netherlands; ^3^ NUTRIM School of Nutrition and Translational Research in Metabolism, Maastricht University, Maastricht, Netherlands

**Keywords:** acnes, neurectomy, long term outcomes, chronic abdominal wall pain, surgical outcomes

## Abstract

**Objective:**

To analyze long-term treatment outcomes of a posterior neurectomy in a large cohort of patients with anterior cutaneous nerve entrapment syndrome (ACNES).

**Summary Background Data:**

The current step-up treatment approach for ACNES involves abdominal wall tender point injections, pulsed radiofrequency, and neurectomy. If an anterior neurectomy fails or pain reoccurs, a posterior neurectomy is considered as a final surgical option. Data on posterior neurectomy treatment outcomes are scarce.

**Methods:**

ACNES patients who underwent a unilateral posterior neurectomy between 2012 and 2022 in a single institution completed a questionnaire regarding their current pain status. Primary outcomes were short- and long-term treatment success, defined as ≥50% pain relief. Patients were stratified whether the operative indication was recurrent pain (>3 months) after an initially successful anterior neurectomy or ongoing pain after an anterior neurectomy.

**Results:**

Data from 260 of 379 patients (77% female, mean age 42 years) were analyzed (68.6% response rate). Sensitivity analysis found that short-term outcomes were similar between responders and non-responders. The recurrent pain group demonstrated significantly better treatment outcomes compared to the ongoing pain group, both in the short-term (7 weeks; treatment success 79.2% vs. 53.2%, *p* < 0.001) and long-term (58 months; treatment success 61.1% vs. 42.0%, *p* = 0.001). Sixteen (minor) complications (6.2%) were reported, resulting in three surgical re-interventions (1.2%).

**Conclusion:**

A posterior neurectomy is long-term beneficial in approximately half of patients although treatment success is better for recurrent pain than ongoing pain. These findings aid in optimizing preoperative patient counseling.

## Introduction

Chronic abdominal pain originates within the abdominal wall in up to 30% of the patients. The cause of abdominal wall pain is often illusive resulting in serious diagnostic and treatment delays. A commonly occurring abdominal wall pain entity is the anterior cutaneous nerve entrapment syndrome (ACNES) [1–3]. The diagnosis is based on previously published major and minor criteria [4], after which a step-up treatment regimen is initiated [1–5]. Treatments starts with injections of local anesthetic agents having a 33% success rate [5, 6]. Patients with ongoing pain may undergo pulsed radiofrequency treatment with approximately 20% success rates [7, 8].

About half of the ACNES patients do not experience satisfactory pain relief after these minimally invasive treatments and almost always opt for surgery [1, 4, 5]. At present, two surgical treatment alternatives are available. An anterior neurectomy involves removing nerves penetrating the anterior fascia of the rectus abdominis muscle sheath. Short- and long term success rates are 70% and 60%, respectively [5, 9, 10]. Patients who experience ongoing pain following an anterior neurectomy or patients who develop recurrent pain are considered eligible for a posterior neurectomy. Which will represent approximately 20% of the entire ACNES population [5, 6, 8–10]. During this operation, a neurectomy of the neurovascular bundle is performed at the level of the posterior sheath of the rectus abdominis muscle [11]. An overall 66% success rate was reported in a group of 41 patients. Interestingly, success rates in patients with ongoing pain was lower compared to patients with recurrent pain >3 months after an initially successful anterior neurectomy (50% vs. 93%) [11].

The aim of the present study was to analyze long-term success rates of a posterior neurectomy in a large cohort of ACNES patients. It was hypothesized that long-term success rates for recurrent pain are superior compared to ongoing pain after an anterior neurectomy. Outcomes will contribute to optimizing preoperative patient counseling and the treatment algorithm for ACNES.

## Materials and Methods

All patients who underwent a posterior neurectomy, identified by a unique surgical procedure code, between January 2012 and January 2022 were eligible for the study. All procedures were performed by four surgeons of SolviMáx, center of excellence for abdominal wall and groin pain, which is a subdivision of the surgical department of Máxima Medical Center, a teaching hospital in the Netherlands. Notably, the vast majority of patients underwent a treatment algorithm including local injections, pulsed radiofrequency, and anterior neurectomy without achieving sufficient pain relief [4, 5, 12]. Consequently, they were counseled for a posterior neurectomy [11].

Patients were excluded if they were unable to read Dutch, were having missing data during follow-up, or were deceased. Patients were also excluded if the unique surgical procedure code was used incorrectly, verified by the operation report (e.g., neurectomy of inguinal nerves, or no posterior neurectomy performed). Furthermore, patients were also excluded if they underwent both an anterior and posterior neurectomy in one session, if they had previously undergone a posterior neurectomy elsewhere, or if a re-do posterior neurectomy was performed at our institute. Lastly, patients with multiple tender points (points of maximal pain) or with bilateral posterior neurectomy were excluded as treatment outcome could not be determined properly.

If patients fulfilled all study criteria, a digital questionnaire was sent by e-mail in December 2023 followed by a reminder after 10 days in case of no response. After 4 weeks, a paper questionnaire was sent if the digital questionnaire was not returned. The questionnaire contained questions regarding pain status shortly after the posterior neurectomy, a potential onset of recurrence of pain (if yes, in proximity of the scar or elsewhere in the abdomen, how long after the surgery it occurred), current average pain score using the Numeric Rating Scale (NRS; range 0–10), and current treatment outcome based on pain relief and patient global impression of change (PGIC) [13]. The translated version of the questionnaire is provided as Supplementary Material S1. Patients were analyzed if completion of the questionnaire was confirmed. The Daily Board of the Medical Ethics Committee (METC) Máxima Medical Center judged that ethical approval was not required for this observational study (METC number: N20.065).

Written informed consent was obtained from the individuals for the publication of any potentially identifiable images.

### Outcome Measures

Baseline patient and pain characteristics including age, gender, body mass index (BMI), baseline NRS, duration of pain, and chronic abdominal wall pain score [14] as well as short-term outcome were extracted from electronic patient records. These baseline data were also used to perform a sensitivity analysis of responders and non-responders to the questionnaire. Treatment success was defined as a ≥50% pain reduction or ≥4 points pain reduction using NRS, while a pain reduction of 30%–50% or ≥2 points using NRS was defined as a moderate success [15]. Conversely, outcome was judged as a failure in case of <30% pain reduction. Pain increase was documented as a separate category. Long-term outcomes were based on the questionnaire. The date of questionnaire receipt was used as follow-up date.

Patients were stratified based on posterior neurectomy indication. Recurrent pain was defined as a minimal 3 month ≥50% pain reduction following an initially successful anterior neurectomy, but with pain recurring in the original area of surgery. Ongoing pain was defined as insufficient or no pain reduction, or a recurrent pain within 3 months after anterior neurectomy.

Primary objectives were short- and long-term treatment outcomes of a posterior neurectomy based on indication for surgery. Secondary objectives included pain recurrence rates, treatment outcomes of additional interventions, treatment satisfaction based on PGIC, and complication rates.

### Posterior Neurectomy Technique

**FIGURE 1:**
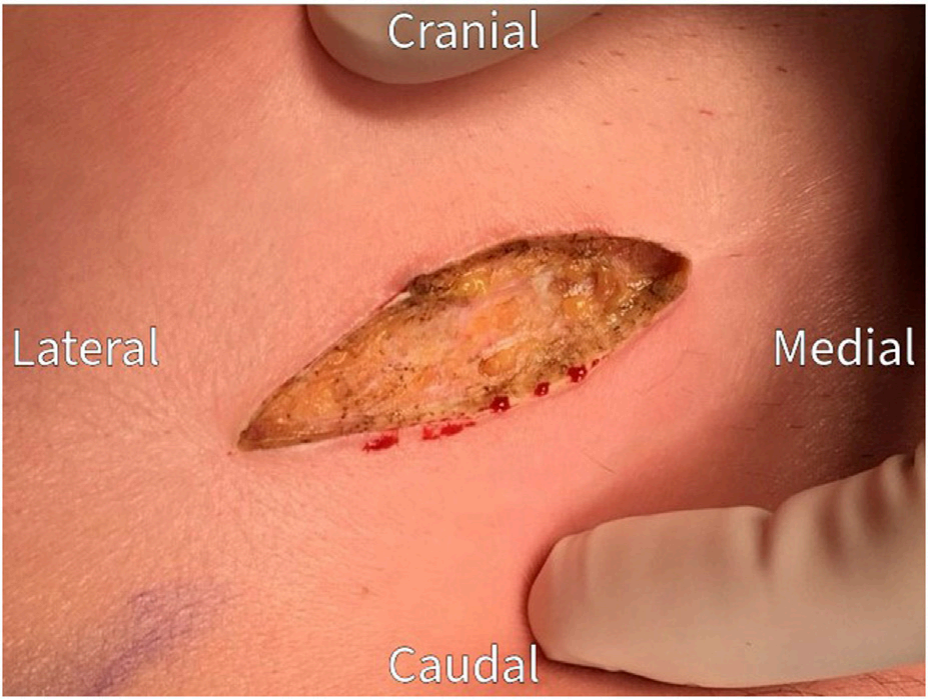
Re-incision of the previous neurectomy scar.

**FIGURE 2:**
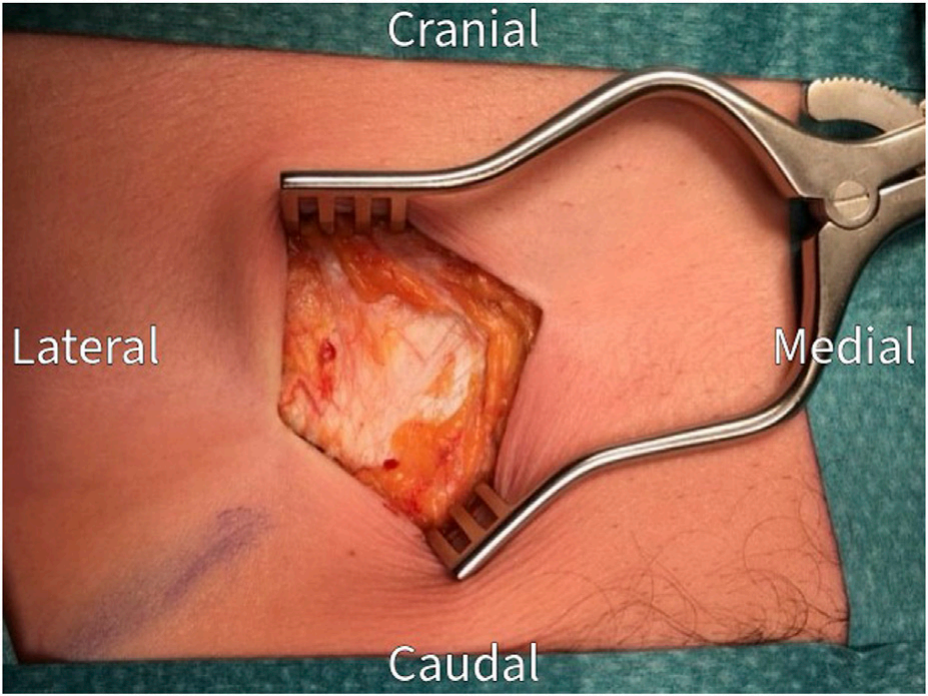
The anterior rectus sheath is exposed after dissection through subcutaneous tissue. Check for and coagulate any remaining penetrating neurovascular bundles.

**FIGURE 3:**
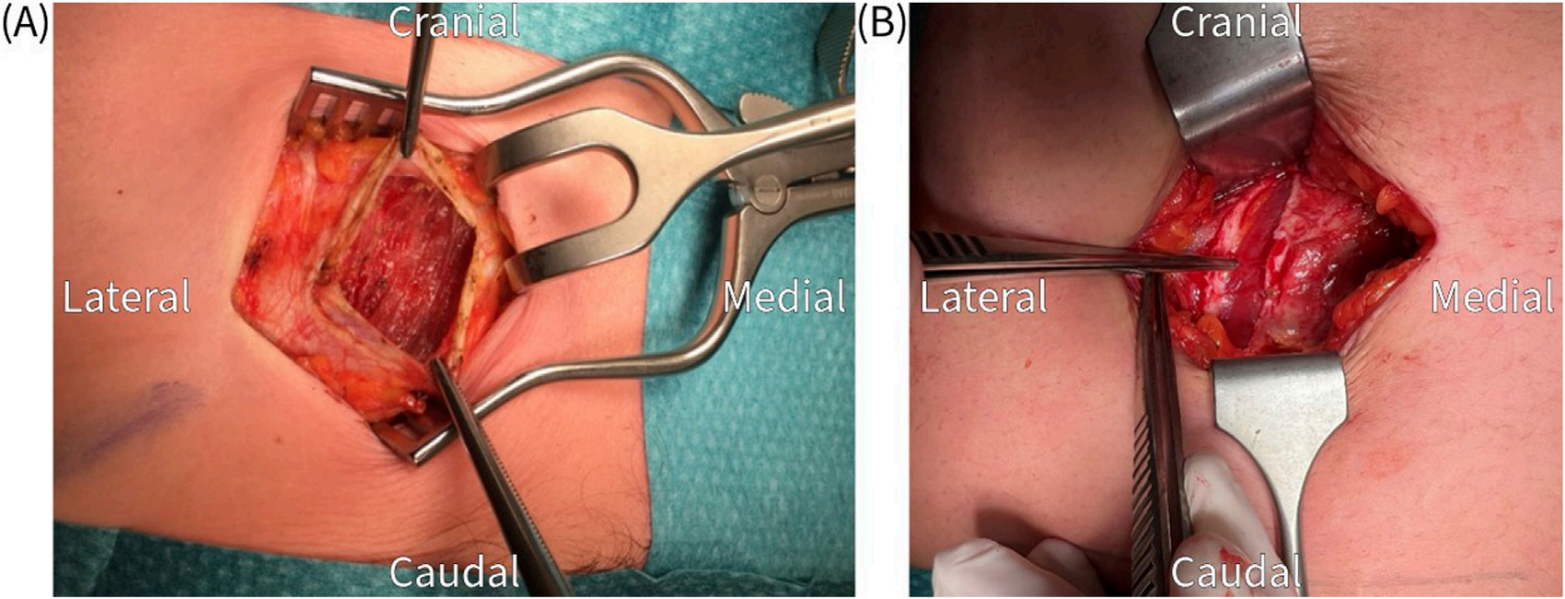
**(A)** Transverse incision of the anterior rectus sheath, which is held up by the tweezers, to expose the rectus abdominis muscle. **(B)** Longitudinal incision of the anterior rectus sheath.

**FIGURE 4:**
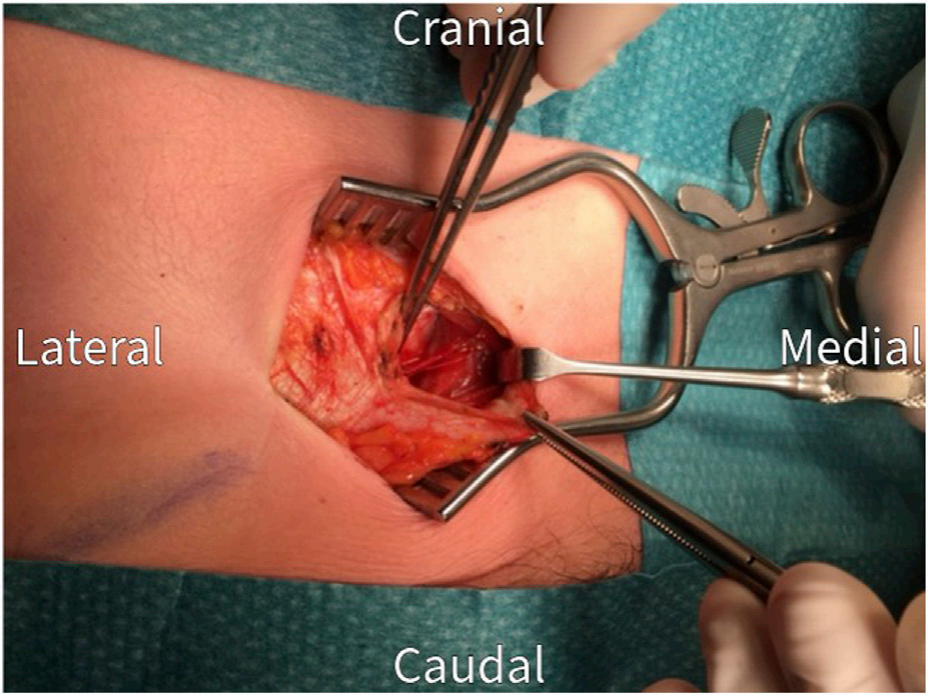
The rectus abdominis muscle is retracted medially to visualize the posterior rectus sheath.

**FIGURE 5:**
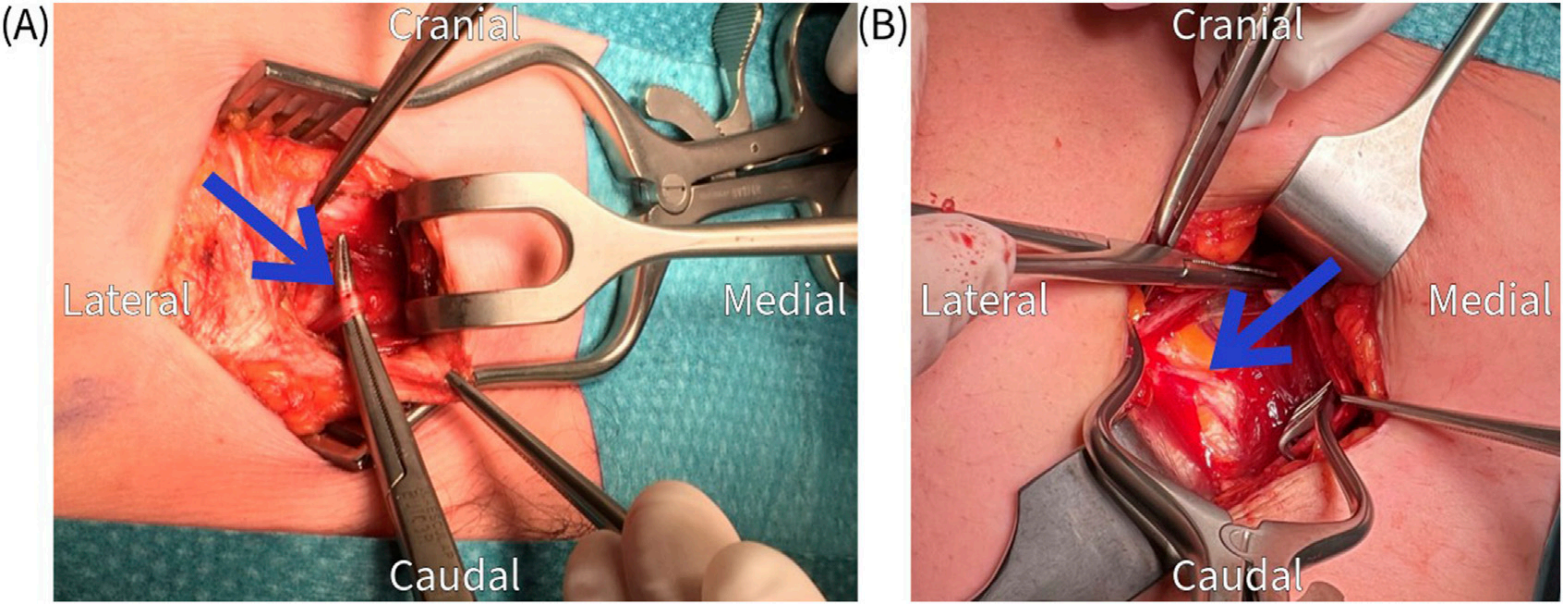
**(A,B)** Identification of an obliquely running nerve (arrow) at the level of the posterior rectus sheath.

**FIGURE 6:**
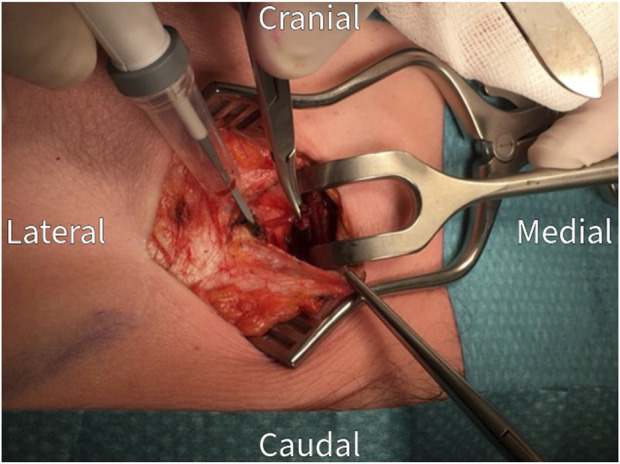
Proximal cauterization and resection of the nerve under traction. Normally at least two nerve bundles are found and excised.

**FIGURE 7:**
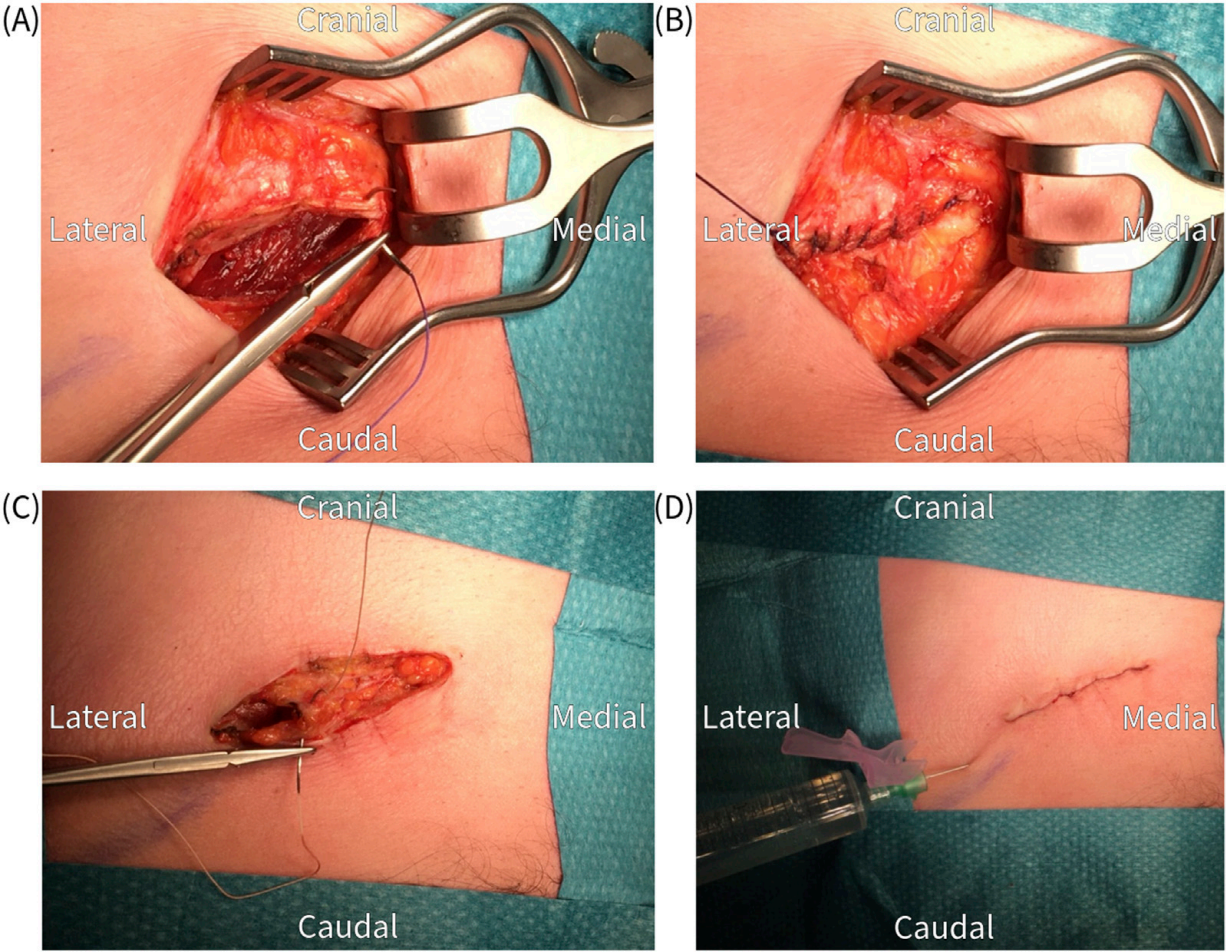
Closure of the wound in layers; **(A,B)** anterior fascia, **(C)** subcutaneous tissue, **(D)** skin and infiltration with a long acting local anesthetic.

The surgical procedure is performed under general anesthesia in a day care setting and lasts approximately 30 min. The anterior neurectomy scar is reopened (Figure 1) and the anterior rectus sheath is identified (Figure 2). Any remaining perforating neurovascular bundles at this level are coagulated (completion of anterior neurectomy). Subsequently, the anterior rectus sheath is opened at its lateral portion through a transverse or longitudinally incision, depending on surgeon’s preference (Figure 3). Rectus abdominis muscle tissue is retracted medially allowing visualization of the posterior rectus sheath (Figure 4). The neurovascular bundle enters this retromuscular space from lateral (of note, depends on the level on the abdominal wall) and passes over the posterior rectus sheath obliquely to enter the rectus muscle from behind (Figure 5). Depending on anatomy, two to three nerves are cauterized or ligated. The destroyed nerve is allowed to retract into the transversus abdominis plane (Figure 6). After infiltration with a long acting local anesthetic agent, the wound is closed in layers (Figure 7).

### Data Analysis

Normally distributed data were presented as means with 95% confidence intervals and were analyzed using the Student’s t-test. Skewed and categorical data were presented as median with interquartile ranges (Q1-Q3) and were analyzed with the Mann-Whitney *U* test. Binary data underwent Fisher’s exact test. Spearman’s rank correlation was used to analyze the correlation between treatment outcomes based on pain reduction and patient’s perception of treatment outcomes based on PGIC. SPSS software Version 22.0 for Windows (IBM, Armonk, NY, United States) was used for the analyses. A *p*-value of <0.05 was considered statistically significant.

## Results

**FIGURE 8:**
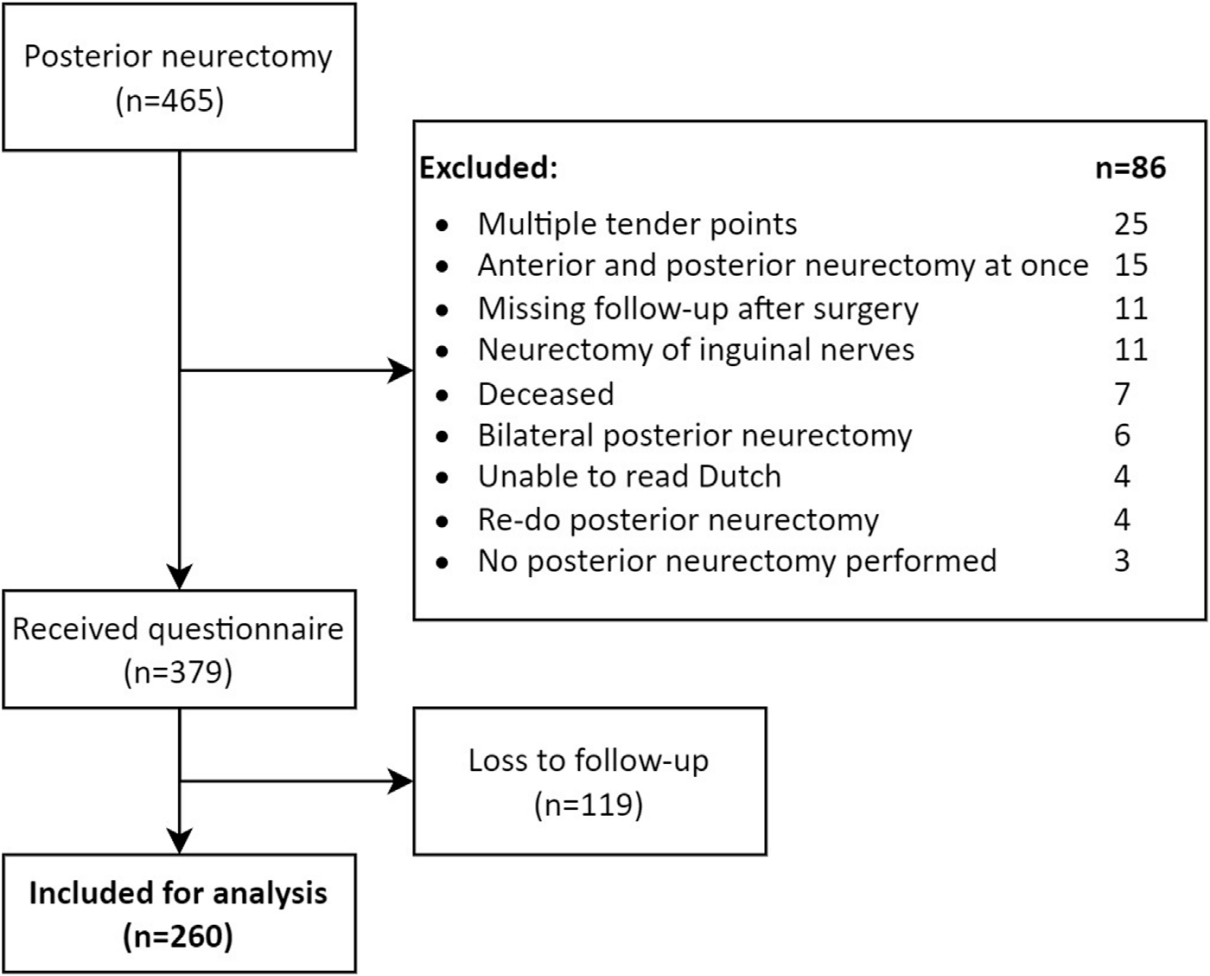
Study flowchart.

A total of 465 patients were registered with the unique posterior neurectomy surgical procedure code during the 10 years of interest. As 86 patients met exclusion criteria, the questionnaire was sent to 379 patients. Eventually, a completed questionnaire of 260 patients was received for analysis [68.6% response rate, (Figure 8)].

The sensitivity analysis revealed that patients who completed the questionnaire (responders) were 4 years older (43 vs. 39, *p* = 0.021) and had a lower pain score before treatment (NRS 7 vs. 8; *p* = 0.006) compared to the group who did not return the questionnaire (non-responders). However, other variables were not different (Table 1).

**TABLE 1:** Responding and non-responding ACNES patients undergoing a posterior neurectomy for recurrent or ongoing pain.

	Responders (n = 260)	Non-responders (n = 119)	*p*-value
Age (yrs)	43 (41–45)	39 (36–42)	**0.021**
Sex ratio, M:F	1:3.3	1:3.6	0.895
BMI (kg/m^2^)	26 (26–27)	25 (24–26)	0.088
NRS before treatment^a^	7 (6–8)	8 (7–8)	**0.006**
Duration of pain (months)^a^	20 (9–42)	18 (9–40)	0.484
Abdominal wall score^a^	14 (12–15)	14 (11–16)	0.523
Indication for posterior neurectomy			0.708
Recurrent pain	72 (27.7%)	30 (25.2%)	
Ongoing pain	188 (72.3%)	89 (74.8%)	
Short-term treatment outcome			0.153
Success	157 (60.4%)	61 (51.3%)	
Moderate success	27 (10.4%)	19 (16.0%)	
No effect	72 (27.7%)	36 (30.3%)	
Increase in pain	4 (1.5%)	3 (2.5%)	

Presented as means with 95% confidence interval or absolute count with percentages.

^a^ Median with interquartile range (Q1-Q3).

BMI, body mass index; NRS, numeric rating scale.

Significant *p*-values are indicated in bold.

### Indication for Posterior Neurectomy

A total of 72.3% (n = 188/260) of the patients received a posterior neurectomy because of ongoing pain after anterior neurectomy and the remaining 27.7% (n = 72/260) for recurrent pain. The latter group had a median pain free period of 8 months [5–12] after an initially successful anterior neurectomy.

### Short-Term Treatment Outcome After Posterior Neurectomy

**FIGURE 9:**
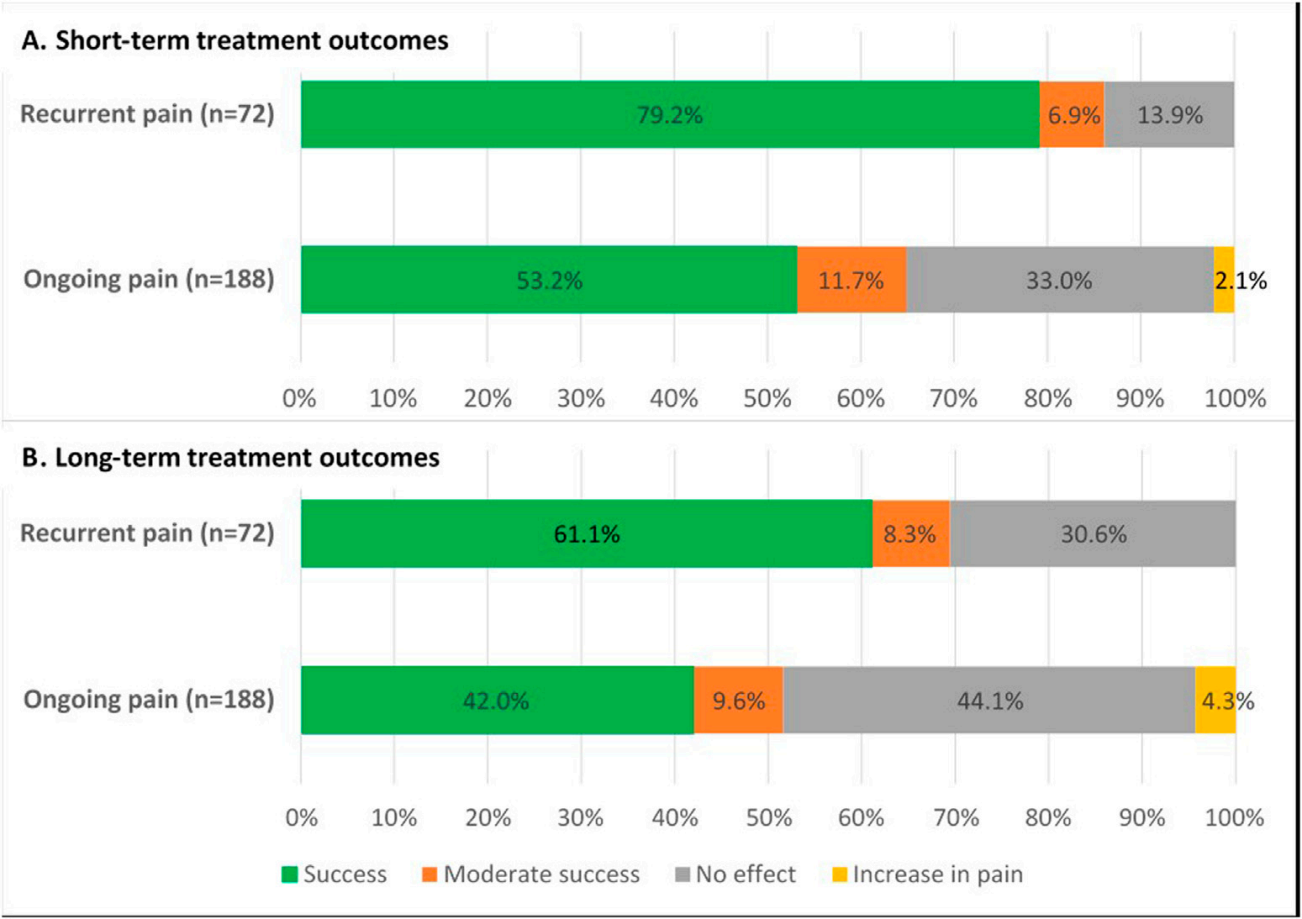
Treatment outcomes of posterior neurectomy for both ACNES groups **(A)** after 7 weeks short-term follow-up (*p* < 0.001) **(B)** after 58 months long-term follow-up (*p* = 0.001).

After a mean follow-up of 7 weeks, short-term treatment success was significantly higher in patients with recurrent pain compared to patients with ongoing pain (79.2%, n = 57/72% vs. 53.2%, n = 100/188; *p* < 0.001). An increase in pain was reported in 2.1% (n = 4/188) of patients with ongoing pain but not in any of the patients with recurrent pain (Figure 9A).

### Long-Term Outcome After Posterior Neurectomy

During a mean follow-up of 58 months (range 20–149), a similar pain recurrence rate after posterior neurectomy was reported in both groups (recurrent pain 22.8%, n = 13/57 vs. ongoing pain 24.0%, n = 24/100; *p* = 1.000). The median pain free period before recurrence after posterior neurectomy in all these 37 patients was 8 months (4–36). Considering these data, long-term treatment outcome remained significantly better in the recurrent pain group compared to the ongoing pain group (61.1% vs. 42.0%; *p* = 0.001) (Figure 9B).

Of the 37 patients with recurrent pain after posterior neurectomy, 20 (54.1%) underwent a re-do posterior neurectomy (9 recurrent group and 11 ongoing group). Reported treatment success was 95.0% (n = 19/20). Seven of the remaining 17 patients (41.2%) became pain free after alternative, less invasive treatment modalities such as pharmacotherapy, manual therapy, injection therapy, or various other treatments by pain specialists.

### Patient Global Impression of Change at the End of Follow-Up

**FIGURE 10:**
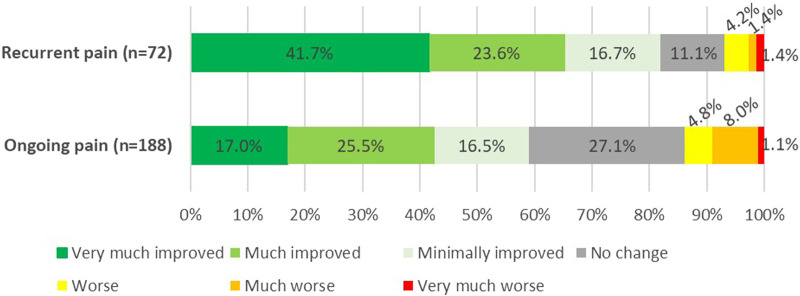
Patients global impression of change (PGIC) following a posterior neurectomy for ACNES at 58 months follow-up (*p* < 0.001).

Although the PGIC methodology reflects the patient’s point of view of change of pain following all received treatments, patients in the recurrent pain group experienced significantly more improvement compared to the ongoing pain group (Figure 10). A strong correlation (R_s_ = 0.843, *p* < 0.001) between PGIC outcome and treatment success based on pain reduction was observed.

### Complications

A 6.2% (n = 16/260) surgical complication rate was observed. Hematoma formation was observed in eight patients, one of which underwent a surgical evacuation. A total of four abscesses were diagnosed with surgical drainage of two. Two patients received antibiotics for superficial wound infections. Another two patients reported wound dehiscence which was treated conservatively. Two patients (0.8%) noticed a persistent bulging of the abdominal wall in proximity to the scar.

## Discussion

The present study is the first to describe short- and long-term treatment outcome of a posterior neurectomy in a large group of ACNES patients. While the posterior neurectomy is a reasonably successful procedure in most patients in itself, outcome of an earlier anterior neurectomy is a crucial factor determining success. Patients with recurrent pain have an 80% short-term and 60% long-term success rate after a posterior neurectomy. In contrast, patients with ongoing pain after an unsuccessful anterior neurectomy have a 50% short-term and 40% long-term success rate. These findings contribute to optimization of the counselling process prior to planning a posterior neurectomy.

To date, just one small study by our team in 41 patients reported on treatment outcome of a posterior neurectomy [11]. A similar significant difference in short- and long-term success rate between both indication groups was found (90% for recurrent pain and 50% for ongoing pain). However, the current study with a much longer follow-up covering a large number of patients is more representative of the whole ACNES population. An acceptably low 6% complication rate with a 1.2% surgical re-intervention rate is similar to previously reported complication rates of the posterior and anterior neurectomy [10, 11]. In the long-term, bulging of the ventral abdominal wall was reported in two patients. This bulging probably results from the loss of motor innervation and consequent atrophy of the rectus abdominis muscle although the fascia remains intact. Therefore, this clinical picture should not be confused with an incisional hernia [16]. Although a substantial portion of studies on ACNES is reported by our center of expertise, we make a vigorous effort to select different patient cohorts for each study. However, there is a possibility of some overlap in patients when addressing different research questions.

The 23% pain recurrence rate of a posterior neurectomy is slightly higher than recurrence rates that were observed after (the first) anterior neurectomy (16%) [10]. This difference may possibly be attributed to the much longer follow-up time (mean 32 vs. 58 months). Another potential explanation is the neurectomy technique. It has been suggested that burying a nerve ending into a muscle has favorable outcomes regarding neuroma prevention and treatment compared to simple neurectomy [17, 18]. For example, during a posterior neurectomy, nerve endings may retract into the transversus abdominis plane. During an anterior neurectomy, nerve endings may retract into the rectus abdominis muscle [11].

A relatively low long-term treatment success rates of 60% and 40% in both groups highlight the complexity of the phenomenon of ongoing pain after anterior neurectomy. Ideally, improved patient selection criteria should prevent unsuccessful surgery, but further research on risk factors or alternative treatment options is also necessary. However, we believe that surgery should not be denied to these patients notwithstanding the relatively low treatment success rates. Severe neuropathic pain as documented in these patients (median NRS of 7) is notoriously difficult to treat and other (non-invasive) treatments, such as pharmacotherapy are associated with similar or even lower success rates [19, 20]. Moreover, most patients already received a variety of treatments before referral to our expertise center indicating an unfavorable case-mix. One could even argue whether the diagnosis ACNES was correct in some of these patients, given the fact that ACNES is a clinical diagnosis, lacking a diagnostic gold standard. Additionally, the pathophysiology of ACNES remains largely unknown. A better understanding of this underlying mechanism is likely to lead to improved treatment options. Further research addressing both diagnostic accuracy and pathophysiology is warranted.

Although the study design potentially harbours a selection (non-responder) bias, the relatively high 69% response rate and similar short-term treatment outcomes of responders and non-responders are reassuring. The 4 year age difference and dissimilar pre-treatment pain score (NRS 7 vs. 8) are probably not clinically relevant. Additionally, these characteristics are not expected to affect treatment outcome as demonstrated in patients who underwent an anterior neurectomy [21]. Another potential bias is related to recall as pain perception may change over time which could influence pain scores, and therefore, perceived treatment outcomes based on pain scores [10]. However, we also assessed treatment outcome using the PGIC and found a strong correlation between treatment outcome based on NRS and PGIC (R_s_ = 0.843). This strong correlation suggests that NRS is reliable for determining treatment outcome in ACNES. Remarkably, over half of the patients who reported recurrent pain after a successful posterior neurectomy underwent a tertiary neurectomy, which in fact is a re-do posterior neurectomy. Interestingly, a 95% treatment success was observed. Further research and data analyses are needed to investigate perioperative findings (e.g., neuroma or remaining neurovascular bundles) and to evaluate how this treatment relates to other treatment options in this specific population.

In conclusion, the outcome of a previous anterior neurectomy needs to be taken into consideration when counseling ACNES patients for a posterior neurectomy. Recurrent pain after an anterior neurectomy is associated with 80% short-term and 60% long-term success rates after posterior neurectomy. A posterior neurectomy should not be denied in patients with ongoing pain after anterior neurectomy, as 50% and 40% short- and long-term success rates are arguably acceptable in chronic neuropathic abdominal wall pain.

## Data Availability

The raw data supporting the conclusions of this article will be made available by the authors, without undue reservation.
