# Supplementary material for: Long-Term Efficacy of Posterior Neurectomy in Anterior Cutaneous Nerve Entrapment Syndrome
Source: J Abdom Wall Surg. 2024 Oct 3;3:13508. doi: 10.3389/jaws.2024.13508 (PMC11484248; doi:10.3389/jaws.2024.13508)
Supplement: Supplementary file 1 [file DataSheet1.PDF]

Survey: Results after a Posterior Neurectomy for ACNES

1. Your previous ACNES treatment consisted of (please check all that apply, multiple answers possible):

   □ One or more abdominal wall injections

   □ PRF treatment via pain clinic

   □ The first surgery (anterior neurectomy)

   □ The second surgery (posterior neurectomy)

   □ I have had more than 2 surgeries

   □ Other, namely: ____

2. On which side did you undergo the second surgery (posterior neurectomy)?

   □ Right

   □ Left

   □ Both sides

3. How satisfied were you with the pain reduction during the first few months after this second surgery (posterior neurectomy)?

   □ Excellent-good: By then I (practically) did not experience any more pain.

   □ Fair-sufficient: By then the pain had decreased by more than 50%.

   □ Moderate: By then the pain had decreased by 30-50%.

   □ Poor: By then the pain was unchanged.

   □ Worse: By then the pain was worse than before.

   □ I don't remember because it was too long ago.

4. At present, are you experiencing ACNES pain ?

□ Yes, after more than 3 months after the posterior neurectomy the pain returned in the same location (near the scar) as before the surgery.

If yes, after how many months ……

□ Yes, after more than 3 months after the posterior neurectomy the pain returned but in a different location.

If yes, after how many months ……

□ Yes, the pain never went away or only went away briefly (< 3 months).

□ No, I no longer have nay ACNES pain.

4. How satisfied are you currently with the pain reduction regarding ACNES symptoms at the site of the second surgery (posterior neurectomy)?

□ Excellent-good: I now have (practically) no more pain.

□ Fair-sufficient: The pain has decreased by more than 50%.

□ Moderate: The pain has decreased by 30-50%.

□ Poor: The pain is unchanged.

□ Worse: The pain is worse than before.

5. What is your current pain score regarding ACNES symptoms at the site of the second surgery (posterior neurectomy) between 0 and 10 ? (circle the correct answer)

No pain Worst imaginable pain
0 1 2 3 4 5 6 7 8 9 10

6. Has your general condition changed since having the second surgery (posterior neurectomy)?

   □ Very much worse

   □ Much worse

   □ Minimally worse

   □ No change

   □ Minimally improved

   □ Much improved

   □ Very much improved

7. After this second surgery (posterior neurectomy), did you develop bulging or an incisional hernia at the site of the scar?

   □ Yes

   □ No

8. Would you like to be kept informed about the results of this survey?

   □ Yes

   □ No

9. If you would like to provide any additional comments, you can do so here.

……….
